# Implementation and review of the care ecosystem in an integrated healthcare system

**DOI:** 10.1186/s12877-023-04146-z

**Published:** 2023-08-24

**Authors:** Michael H. Rosenbloom, Bhavani Kashyap, Ana Diaz-Ochoa, Jan Karrmann, Aleta Svitak, Jennifer Finstad, Ann Brombach, Ann Sprandel, Leah Hanson, Sarah Dulaney, Katherine Possin

**Affiliations:** 1 HealthPartners Center for Memory & Aging, St. Paul, MN USA; 2 HealthPartners Institute, Bloomington, MN USA; 3 Neuroscience Research, HealthPartners Neuroscience Center, 295 Phalen Blvd, St Paul, MN 55130 USA; 4 Memory and Aging Center, University of California, San Francisco, CA USA

**Keywords:** Care ecosystem, Care team navigator, Person with dementia, Case management

## Abstract

**Background and objectives:**

The University of California, San Francisco Memory and Aging Center (UCSF-MAC) led the development and tested a collaborative care model delivered by lay care team navigators (CTNs) with support from a multidisciplinary team known as the Care Ecosystem (CE). We evaluated outcomes related to the feasibility of the CE in a non-academic healthcare system, including acceptability, adoption, and fidelity to the original UCSF model.

**Research Design and methods:**

The CE team at HealthPartners consisted of two CTNs, a social worker, an RN, a program coordinator, and a behavioral neurologist. Intake forms were developed to collect demographic, baseline, and annual data at one year related to dementia severity and caregiver status. Experience surveys were completed at 6 and 12 months by participating caregivers. All data was entered into REDCap.

**Results:**

A total of 570 PWD-caregiver dyads were recruited into the CE: 53% PWDs female, average age 75.2 ± 9.43, 19% living within rural communities. Of the 173 dyads assessed at one year, 30% responded to the annual intake forms and 58% of responded to experience surveys. At one year, PWDs progressed in disease severity and functional impairment, although caregiver burden and mood remained unchanged. We observed a significant reduction in caregiver reported emotional challenges associated with caregiving, sleep problems, and obtaining caregiver help at one year. 86% of caregivers reported feeling supported by their CTN nearly always or quite frequently, and 88% rated the CTN as highly responsive to what was important to them.

**Discussion and implications:**

The CE was feasible and well-received within a non-academic healthcare system.

## Introduction

Alzheimer’s disease (AD) affects 6.2 million individuals in the U.S., representing the most common cause of dementia [1]. In addition to affecting the person with dementia (PWD), AD also impacts caregivers, who provide an estimated 15.3 billion hours of care valued at nearly $257 billion [1].

Despite PWDs having an increased risk for healthcare utilization compared to non-dementia patients [2] the delivery of care for this population has been inconsistent in quality [3]. Gaps in the care of PWDs include lack of support and training for caregivers, poor care transitions, and inconsistent access to community-based services. Care coordination has been shown to improve the behavioral symptoms in PWD and delay long-term care placement in PWDs.

The University of California, San Francisco Memory and Aging Center (UCSF-MAC) led the development and tested a collaborative care model delivered by lay care team navigators (CTNs) with support from a multidisciplinary team, inclusive of dementia expertise in nursing, social work and pharmacy known as the Care Ecosystem (CE). A single-blind, randomized clinical trial consisting of 780 PWDs and their caregivers recruited from academic institutions revealed that the CE improved PWD quality of life while reducing ED visits, caregiver depression, and caregiver burden compared to usual care [4]. The CE is distinct from traditional social worker-based programs in that the healthcare providers who interface most commonly with the patient and caregiver have limited clinical experience and are mentored through an online training program, ultimately reducing the potential cost of these services.

As an early adopter of this cooperative approach, HealthPartners, a non-academic, healthcare delivery organization serving the greater Minnesota and Wisconsin populations began a collaboration in 2018 with UCSF to adapt and test the feasibility of the CE in an integrated health system. Here, we share descriptive data and results from a process evaluation that characterizes lessons learned to inform future iterations of the Care Ecosystem. Our outcomes were most focused on (1) acceptability of the program to caregivers, (2) adoption of the program by our healthcare system, and (3) fidelity to the original CE model.

Since the HP-CMA CE program did not have a control group for comparison, the major focus in terms of outcome was feasibility. Outcomes related to the feasibility of the CE in a healthcare system included survey data described above to represent *acceptability*, the number of dyads enrolled into the CE to address *adoption* by HealthPartners, and the number and types of adaptations compared to the original CE to address *fidelity* to the original UCSF model.

## Methods

### Implementation and design

The CE intervention was incorporated into a neurology-based memory clinic-HealthPartners Center for Memory and Aging (HP-CMA) and was approved by the department chair. An integrated, fee-for-service, healthcare system, HP is an insurance provider to 1.8 million insurance members and provides care delivery to 1.2 million patients. The HP-CMA CE team consisted of two CTNs, a social worker, an RN, a project coordinator, and a behavioral neurologist. Two CTNs functioned as case managers and had the most direct contact with PWDs and caregivers, providing telephonic support mainly to the caregivers. One CTN had immigrated from Venezuela, obtained an MPH, and participated in prior dementia clinical support services for the Latinx community whereas the other CTN had previously worked as an administrative assistant with little experience working with PWDs. A social worker advised CTNs and provided consultation for more complex patients. An RN assisted with any questions related to prescription medications and side-effects as well as facilitated direct communications with the neurologist caring for patients and caregivers. The CE coordinator was responsible for training CTNs, coordinating meetings, organizing patient-centered materials, and ensuring data integrity. The behavioral neurologist provided oversight and clinical guidance related to the CE team. Due to resource limitations, the HP-CMA CE lacked having the pharmacist as included in the UCSF CE [4].

All CTNs completed the freely available training program on the Canvas Network that includes over 30, ten minute didactic videos with accompanying 3–5 question quizzes intended to reinforce learning. The didactic videos cover topics such as geriatric syndromes, an overview of common types of dementia, supporting caregivers, insurance and public benefits, medications, advance care planning, end of life care, and non-pharmacological behavior symptom management. The Care Ecosystem training is intended to supplement site specific training and clinical observations, written care protocols and educational materials, and ongoing clinical supervision through weekly case discussions. Four neurology providers referred dyads to the CE based on having a dementia or MCI diagnosis. Intake forms were developed to collect baseline and annual l data to evaluate dementia severity and caregiver status. Baseline demographic data were collected based on race, ethnicity, and rural versus non-rural status. Rural was defined by living greater than 30 miles from an urban dementia subspecialty clinic. Experience surveys were completed every 6 months by participating caregivers. A data manager with a research background who was not involved with CE care delivery team was assigned to supervise the REDCap database.

From January 2019 to October 2021, a total of 570 PWDs and caregiver dyads were recruited from the HP-CMA dementia clinic. Dyads were recruited into the CE if PWDs were aged ≥45 and had a diagnosis of dementia or MCI. Caregivers either received a single session of telephonic support **(CE Lite)** or full support from the CE team (calls ranging from daily to quarterly based on need) **(CE Full)** based upon assessment of need. If dyads expressed concerns regarding multiple caregiver needs, safety, management of neuropsychiatric symptoms, navigating community resources, or had limited access to dementia support, they were entered into the CE Full. Those dyads lacking any significant caregiver concerns related to neuropsychiatric symptoms, safety, and had good access to resources were entered into CE Lite. Dyads originally enrolled in CE lite would be transitioned to CE full if caregiver needs required increasingly frequent calls (e.g. >1 call/month) or based on clinical judgment. Weekly meetings were held with the CE team to discuss complicated cases that were particularly challenging from a psychosocial perspective. All data related to demographics, dementia severity, caregiver wellbeing, caregiving needs, and healthcare utilization were entered into a REDCap database. All care plans and clinical encounters were also entered into the electronic medical record (EMR) by the CE team.

### Data analysis

#### Measurement of adoption

Data were collected through mailed or online intake forms at baseline and then repeat questionnaires at 12-months by a subset participating caregivers (n = 173). Dyads were contacted by phone if surveys went uncompleted. Caregivers were responsible for completion of intake forms. Once the intake form was completed, the dyad was officially enrolled and contributed to the total enrollment number, which represented adoption of the CE program. The data captured was modeled after outcomes described by Possin and colleagues. These outcomes included frequency of ED visits/hospitalization for PWD, caregiver depression score on the Patient Health Questionnaire (PHQ)-9 (minimal depression 0–4; mild-moderate 5–14; severe > 14) [5] and caregiver burden score using the 12 item Zarit burden score, a subjective evaluation of caregiver burden that been translated into multiple languages [6] All of the above scales were include in the baseline and 12 month follow-up sent to caregivers.

#### Measurement of acceptability

CTNs were evaluated by dyads (PWD and caregiver) through experience surveys that were either mailed or electronically sent to participants and included questions related to the experience (eg. “Do you feel listened to by your CTN?”). Participants could select the following options, which were based on patient satisfaction questionnaires: “never,” “rarely,” “sometimes,” “quite frequently,” and “nearly always” with the latter two representing a clinically favorable response.

#### Measurement of fidelity

Fidelity to the UCSF CE model was assessed qualitatively be compared the HP-CMA CE to the original model in terms of the CE team participants, the “dose” of the intervention (e.g. Frequency and duration of phone calls from CTN to caregiver), the data gathering process, and outcome measures included on the baseline and one year follow-up intake forms.

A retrospective review of data captured by REDCap and the EMR was performed by both CTNs and research support staff to record the number of ED visits and hospitalizations for PWDs along with factors contributing to healthcare utilization. Elective procedures were excluded. Chief complaint for ED visits and hospitalizations were reviewed from the EMR by a behavioral neurologist.

This study included dementia severity measures (dementia severity rating scale-DSRS and functional assessment questionnaire-FAQ). The DSRS is a brief informant-rated, multiple-choice questionnaire made up of 12-items that measure functional abilities in persons with cognitive disorders (score 0–18 mild; 19–36 moderate; 37–54 severe) [7] whereas the FAQ is a brief subjective assessment of instrumental activities of daily that is typically completed by the caregiver with scores ≥9 suggestive of dementia [8, 9].

Finally, information was collected relating to medication safety at baseline and one year: the number of participants using a pillbox to assist with medication, the number of participants using 10 or more medications, and the number of participants taking blood thinners.

Within intake forms distributed to caregivers, there were questions related to caregiver immediate and anticipated needs and caregiver safety concerns. We further included outcomes related to advance care planning– completion of advance directives, durable power of attorney, completion of a POLST, and interest in palliative care.

Univariate analysis was performed using appropriate tests. The mean (standard deviation), median (IQR) were reported for continuous variables, while percentages were reported for the categorical variables. Paired t-tests, χ^2^ test and fisher exact tests were utilized in comparing the groups. Missing data was identified and highlighted in the tables specific to each variable analyzed. Patients with missing data were excluded only for analysis of the specific variable but included in other analyses. No sensitivity analysis or confounding factors were addressed. Statistical analysis was performed using R statistical program, version 3.5.1 [10]. All p-values were 2 sided with statistical significance at 0.05.

## Results

### Demographics

A total of 570 (84% full; 16% lite) dyads were enrolled into the HealthPartners CE and completed baseline intakes between January 2019 and October 2021 (Table 1). One-year follow-up intake forms were completed by n = 173 (30.4%) dyads participating in the CE. Compliance in completion of these documents was inversely related to the severity of the illness as rated by the DSRS: mild stage (45.2% completion), moderate stage (31.0%), and severe stage (13.8%). Non-responders” had a mean baseline DSRS 20.66±9.7, FAQ 20.07±7.9, Zarit Burden 18.8±10.0, and PHQ of 4.2±4.3. Furthermore, non-responding dyads were more likely to have PWDs with higher DSRS, FAQ scores and caregivers with higher Zarit burden scores. With respect to survey evaluations, 58% of caregivers responded at 12 months, providing data about the CE experience.

**Table 1:** Care Ecosystem Participant Demographics

	Total Enrolled N = 570	Completed Intake and Year 1 N = 173
Enrollment Type		
Full	477 (83.7%)	154 (89%)
Lite	93 (16.3%)	19 (11%)
Location		
Urban	463 (81.2%)	141 (81.5%)
Rural	107 (18.8%)	32 (18.5%)
**Persons Living with Dementia**		
Age in Years (Mean ± Standard Deviation) ^a^	75.15 ± 9.43	72.17 ± 9.18
Gender		
Male	301 (46.7%)	87 (50.3%)
Female	344 (53.4%)	85 (49.1%)
Other	1 (0.6%)	2 (0.4%)
Race^b^		
White-Non-Hispanic	410 (71.9%)	155 (89.6%)
White Hispanic	32 (5.6%)	9 (5.2%)
Asian	13 (2.3%)	3 (1.7%)
Black or African American	17 (3%)	4 (2.3%)
Native Hawaiian or Other Pacific Islander	1 (0.2%)	0 (0%)
American Indian or Alaska Native	1 (0.2%)	0 (0%)
Two or more races	3 (0.5%)	1 (0.6%)
Other	9 (1.6%)	1 (0.6%)
Ethnicity^c^		
Hispanic or Latino	28 (4.9%)	10 (5.8%)
Not Hispanic or Latino	417 (73.2%)	154 (89%)
Hmong	2 (0.4%)	0 (0%)
African	12 (2.1%)	4 (2.3%)
**Care Partner**		
Age in Years (Mean ± Standard Deviation) ^d^	66.39 ± 13.48	65.15 ± 13.02
Gender		
Male	149 (26.1%)	52 (30.1%)
Female	349 (61.2%)	119 (68.8%)
Other	2 (0.4%)	1 (0.6%)
Race^e^		
White-Non-Hispanic	422 (74%)	150 (86.7%)
White Hispanic	31 (5.4%)	10 (5.8%)
Asian	16 (2.8%)	4 (2.3%)
Black or African American	17 (3%)	3 (1.7%)
Native Hawaiian or Other Pacific Islander	0 (0%)	0 (0%)
American Indian or Alaska Native	2 (0.4%)	2 (1.2%)
Two or more races	5 (0.9%)	2 (1.2%)
Other	5 (0.9%)	1 (0.6%)
Ethnicity^f^		
Hispanic or Latino	32 (5.6%)	11 (6.4%)
Not Hispanic or Latino	423 (74.2%)	153 (88.4%)
Hmong	2 (0.4%)	0 (0%)
African	10 (1.8%)	2 (1.2%)
Relationship to Patient^g^		
Spouse	277 (48.6%)	105 (60.7%)
Daughter	141 (24.7%)	39 (22.5%)
Son	37 (6.5%)	10 (5.8%)
Partner	11 (1.9%)	5 (2.9%)
Sibling	18 (3.2%)	8 (4.6%)
Friend	6 (1.1%)	2 (1.2%)
Other Family	16 (2.8%)	3 (1.7%)
Neighbor	0 (0%)	0 (0%)
Hired Caregiver	0 (0%)	0 (0%)
Education^h^		
Less than High School	11 (1.9%)	2 (2%)
High School or GED	32 (5.6%)	8 (4.6%)
Some College/No Degree	85 (14.9%)	37 (21.4%)
Associates Degree	30 (5.3%)	11 (6.4%)
Bachelor’s Degree	166 (29.1%)	58 (33.5%)
Master’s Degree	112 (19.6%)	33 (19.1%)
Doctorate	38 (6.7%)	18 (10.4%)

^a^No data available – 2 in Total enrolled; ^b^ No data available – 84 in Total Enrolled; ^c^ No data available – 111 in Total enrolled and 5 in Completed Intake and Year 1; ^d^No data available – 283 in Total enrolled and 28 in Completed Intake and Year 1; ^e^ No data available – 72 in Total Enrolled and 1 in Completed Intake and Year 1; ^f^ No data available – 103 in Total enrolled and 7 in Completed Intake and Year 1; ^g^ No data available – 64 in Total enrolled and 1 in Completed Intake and Year 1; ^h^ No data available – 96 in Total enrolled and 7 in Completed Intake and Year 1.

A total of 139 dyads or 80% of those completing year one forms filled out online one year intake forms as opposed to phone intake. 53% of patients were female and 47% were male with the average age being 75.2 ± 9.43 (Table 1). Baseline DSRS [7] showed 65% mild, 33% moderate, and 2% severe dementia. On the FAQ [9] 13% of PWDs had possible mild cognitive impairment and 87% were functionally impaired. During the CE project, 89 patients (15.6%) died. The mean number (SD) of calls from CTNs was 6.95 (4.15) (median, 6; interquartile range, 4–9 calls) and call length 16.37 (15.01) (median, 12; interquartile range 6–12 min) as calculated per dyad/year.

For caregivers, 61.2% of participants were female with the average age being 66.39 ± 13.48. A majority of caregivers were either spouses (48.6%) or children (31.2%).

### CE sustainability and feasibility

Between January 2019 and October 2021, a total of five CTNs completed the CE training program. Of these, three CTNs transitioned out of their positions within a 6-month period to pursue graduate degrees. Four separate individuals provided nursing support to the CE from January 2019 to October 2021. Nurses hired to perform telephone triage for the HP-CMA served in this role. During the early phase of the COVID-19 pandemic in 2020, three nurses left the position at HP-CMA for roles outside the health system. The social worker role was fulfilled by three separate individuals.

### Dementia progression & caregiver wellbeing

**Fig. 1:**
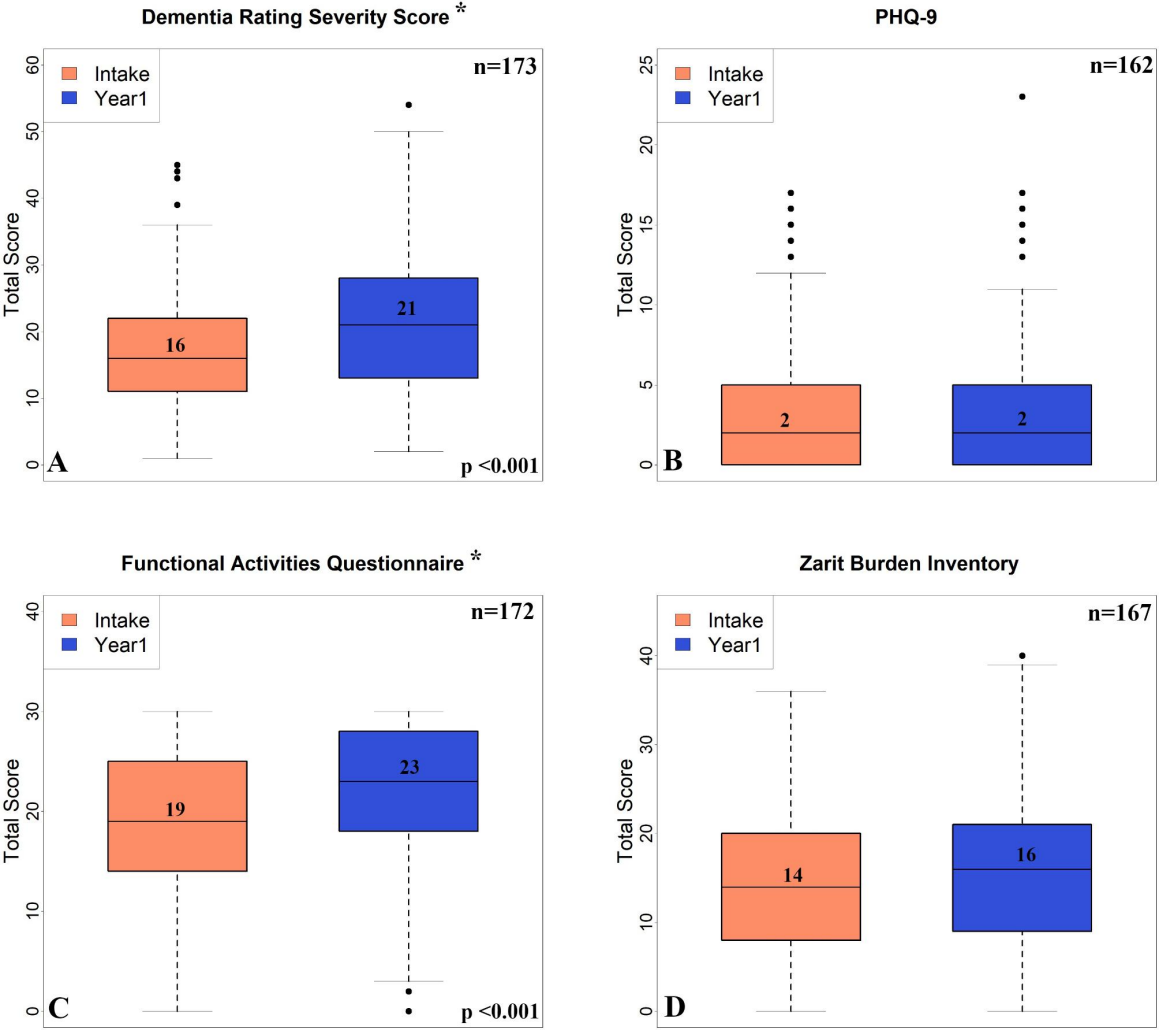
Change in Measures of Dementia Progression and Caregiver Wellbeing. Box plots of total scores of the following measures at intake and year 1 - (A) Dementia Rating Severity Score (DSRS); (B) Functional Activities Questionnaire (FAQ); (C) PHQ-9 and (D) Zarit Burden Inventory. * p < 0.001 between intake and year 1 using paired t-test. Numbers in the middle of the boxplot represents the median for each of the measures

As expected, measures of dementia severity and functional impairment in the PLWD significantly increased during the one-year period as measured by the DSRS (16→21, p≤0.05 and FAQ (19–23, p≤0.05) (Fig. 1). Despite disease progression, caregiver outcomes for PHQ-9 and Zarit Burden Inventory were unchanged between baseline and year one. Caregivers with moderate-severe depression mostly experienced a qualitative reduction in the PHQ9 score at year 1. No significant differences in dementia severity and caregiver burden-related outcomes were found when comparing rural to non-rural groups. There was no relationship between mean call duration/# of calls and the Zarit burden score.

### Caregiver needs & patient safety

**Fig. 2:**
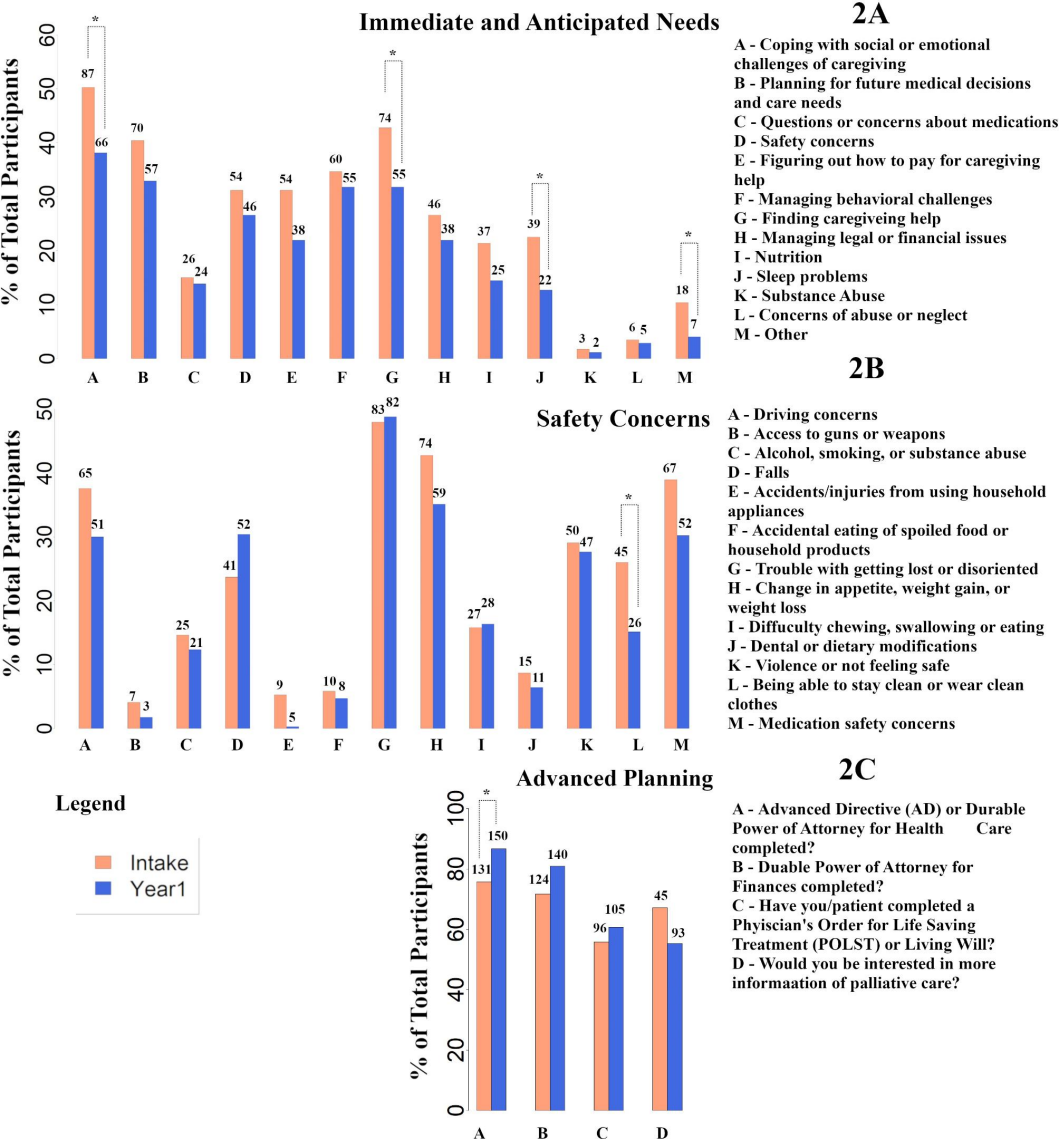
Caregiver Needs and Patient Safety. Bar plots of survey questionnaire results administered to assess caregiver immediate and anticipated needs (2 A), safety concerns (2B) and advanced planning (2 C) at intake and year 1. X-axis represents the percentage of the total number of respondents (includes only data from participants who responded for the specific question). * p < 0.05 between intake and year 1 using Fisher’s exact test. Numbers above the bars represent participants who responded as “yes” for the specific question

Compared to baseline, all caregiver immediate and anticipated needs decreased after the one-year period (Fig. 2a). There was a significant reduction from baseline in percentage of participants answering “yes” for the following: coping with social/emotional challenges of caregiving (from 87→66, p≤0.05), finding caregiving help (74→55, p≤0.05), and sleep problems (39→22, p≤0.05) after one year of CE participation. Likewise, all safety concerns (Fig. 2b) decreased compared to baseline, but only the ability to stay clean or wear clean clothes (45→26, p≤0.05) remained significant after statistical analysis. No significant differences in outcomes were found when comparing rural to non-rural groups nor was there a relationship between the dose of the intervention, measured by mean call duration/# of calls, and caregiver-related outcomes.

### Healthcare utilization and medical care

**Fig. 3:**
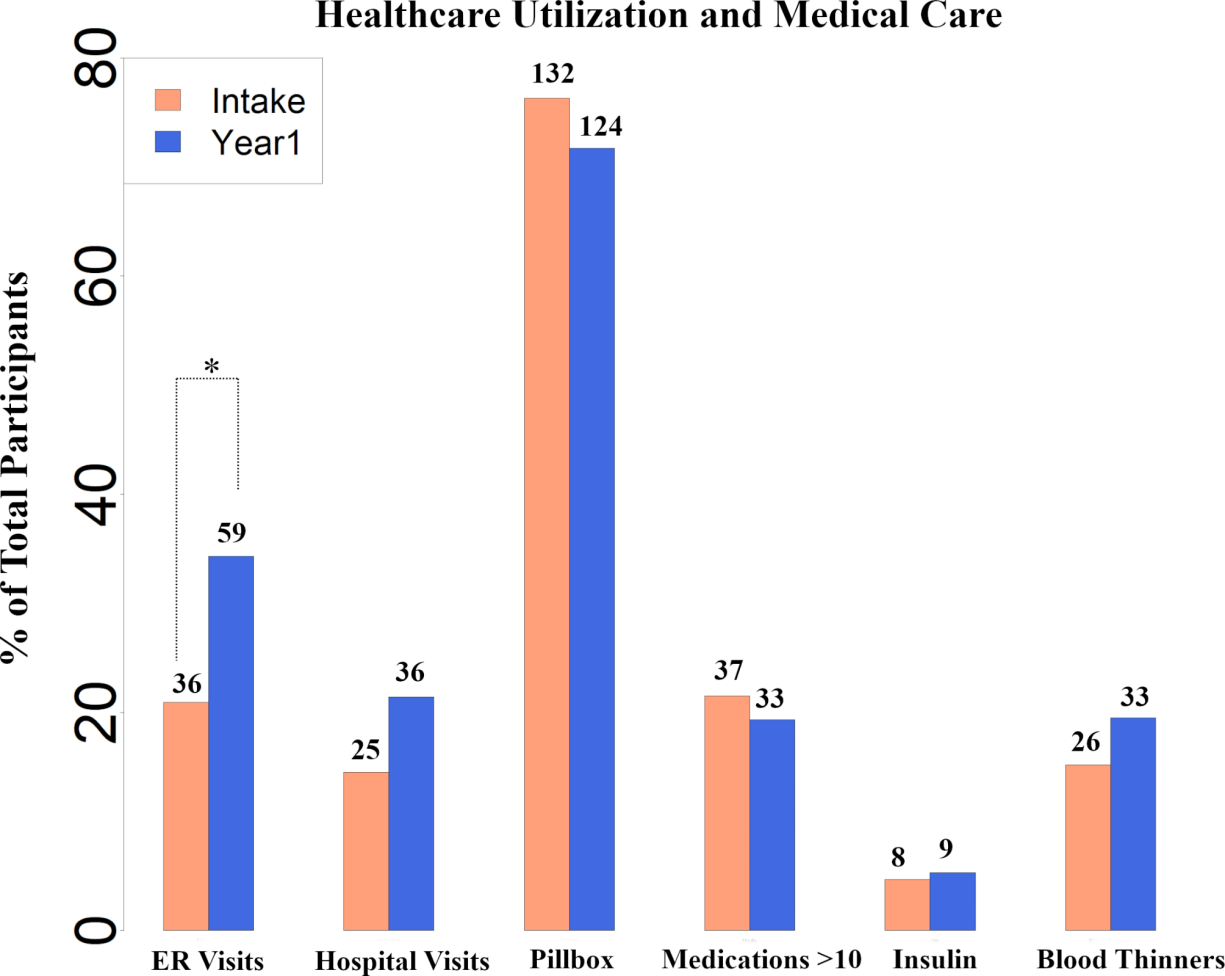
Healthcare Utilization and Medical Care. Bar plots of survey questionnaire results at baseline and 12 months for the following: (1) emergency room visits; (2) overnight hospitalization; (3) utilizing pill box; (4) taking more than 10 medications; (5) prescribed insulin; (6) prescribed blood thinners at intake and year 1. X-axis represents the percentage of the total number of respondents (includes only data from participants who responded for the specific question). * p≤0.05 between intake and year 1 using Fisher’s exact test. Numbers above the bars represent participants who responded as “yes” for the specific question

Longitudinal outcomes related to hospitalizations and ED visits showed that the % of total participants presenting to the ED significantly increased from 36→59, p < 0.05 (Fig. 3) over one year whereas the number of hospitalizations did not significantly change over this time. Approximately, 2.6% of acute care events were related to neuropsychiatric symptoms whereas the majority of events were related to internal medicine-related chief complaints. Healthcare utilization outcomes did not differ between rural and non-rural populations. There was no relationship between the mean call duration/# of calls with the CTN and the number of ED visits/hospitalizations.

Of the 570 dyads enrolled, the number of participants using a pillbox to assist with medication compliance dropped from 132 at baseline to 123. In addition, 37 individuals were taking 10 or more medications at baseline as compared to 33 after 1 year. The number of PWD prescribed blood thinners increased from 26 to 34. None of these changes were found to reach statistical significance.

CE CTNs discussed advance care planning with PWDs and caregivers. This included completion of advance directives, durable power of attorney, completion of a POLST, and interest in palliative care. By the end of year 1, 87% of PWDs had established an advance directive, 81% established a durable power of attorney for finances, 61% had completed a POLST, and 53% expressed interest in palliative care. A significant increase in the number of PWDs completing an advanced directive as represented by completed forms in EMR was found between baseline (131) and year one (150), p≤0.05.

### CE program evaluation

**Fig. 4:**
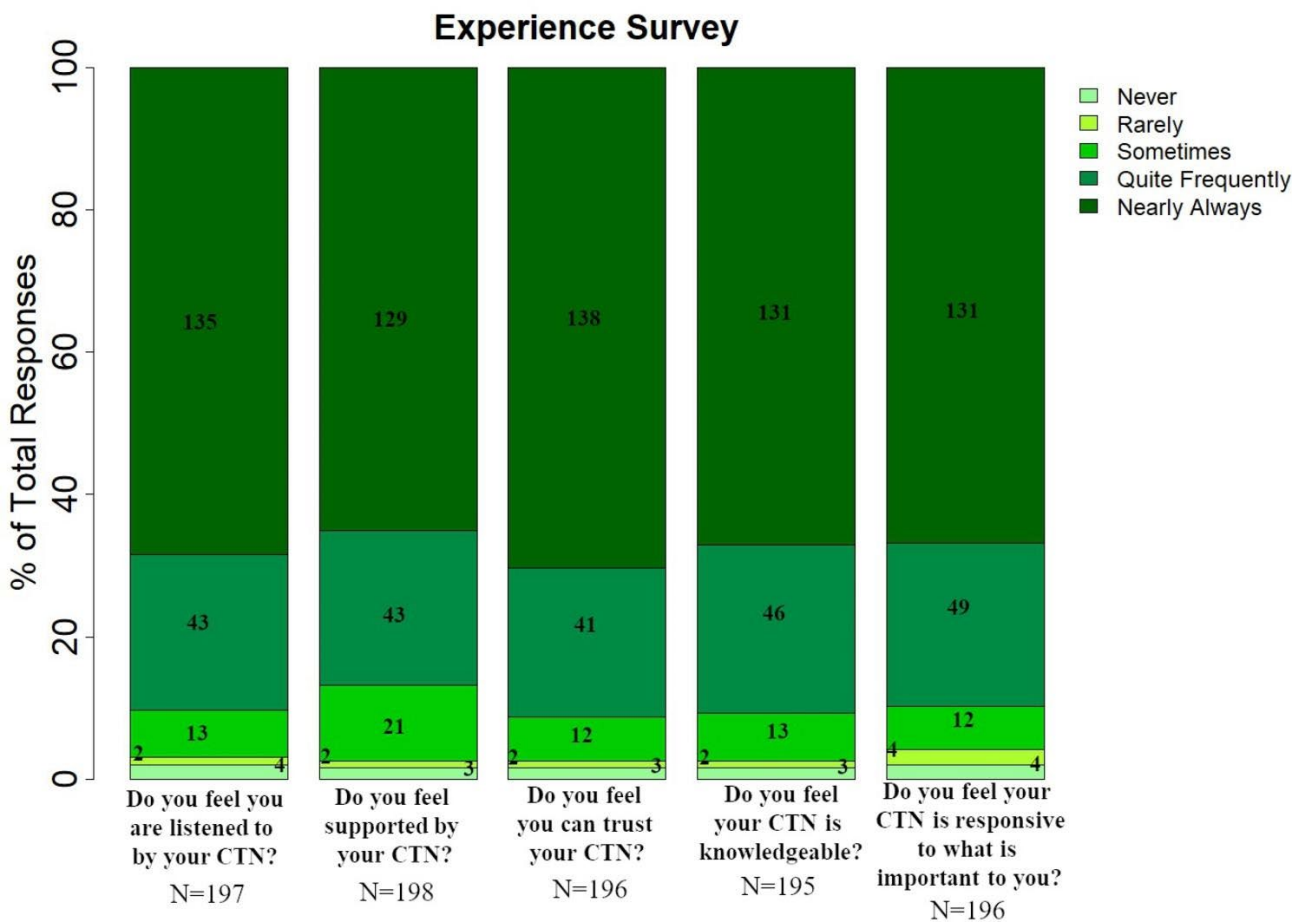
Care Experience. Results of survey administered to assess the care experience of the program assessed at 6 months and 1 year

All participating dyads were asked to complete experience surveys at 6 and 12 months relating to their experience. Of participating dyads, 90% reported that they quite frequently or nearly always felt that their CTN listened to them, they trusted their CTN, and felt that their CTN was knowledgeable (Fig. 4). In addition, 87% of dyads felt they were quite frequently or nearly always supported by their CTN, and 92% reported that their CTN was responsive to what was important to them.

## Discussion and implications

We found that the CE program was feasible in a high-volume neurology clinic over a 34-month enrollment period., and there was acceptability by caregivers based on survey data. The CTNs, despite carrying a relatively larger number of dyads compared to prior studies, were valued by dyads with 87–92% of participants rating their experiences as being favorable. These numbers are similar to what has been documented in the past for CTNs and suggest that PWDs and caregivers appreciate this service regardless of whether the healthcare setting is academic or non-academic [4].

In terms of adoption by our healthcare system, the two HP-CMA CTNs were able to take on a larger caseload compared to the literature, managing 570 referrals to the program. Compared to the original UCSF CE model, our ‘dosage’ of CTNs calls was less than that of the published trial (median number of calls were 6 versus 12, and median duration was 12 min versus 40) [4]. However, it should be noted that 65% of patients of PWD at baseline were considered “mild” stage (2% advanced stage) based on the DSRS whereas the UCSF CE as described by Possin and colleagues consisted of 49.8% mild stage patients (21.5% advanced stage), thus suggesting that the HP-CMA cohort was less complicated and therefore, required fewer calls from CTNs.

Only 30% of dyads managed to complete the annual intake form at 12 months despite all participants receiving phone call reminders from the CTNs. Based on the data, severity of dementia was a factor that impact the completion of annual intake forms. Furthermore, we suspect that the length of the intake form may have impacted our ability to collect outcome data on a majority of participants. Thus, this investigation was not powered nor was it designed to compare healthcare-related outcomes between those PWDs enrolled and not enrolled in the CE.

Nevertheless, our preliminary analysis did reveal a variety of interesting findings. The CE resulted in significant benefit in helping caregivers cope with the emotional challenges of caregiving, address sleeping problems, and assist with finding caregiving help as shown in Fig. 2. It seemed that caregivers with moderate-severe depression experienced a reduction of the PHQ9 over the 1-year period. In addition, CTNs assisted dyads with proactive planning such that there was a significant increase in the numbers of PWDs completing an advance directive or durable power of attorney. Completion of advanced directives is frequently overlooked by PCPs and specialists with only 7.4–42.3% of PWDs completing advanced directives [11].

Results at one year relating to medication safety were counter-intuitive and deserve further explanation. The number of participants using a pillbox decreased, the number of PWDs taking 10 or more medications increased, and there were an increased number of PWDs prescribed blood thinners at one year compared to baseline intake. One explanation is that our program did not have the clinical infrastructure to support a dedicated pharmacist as described in the original CE program. As a result, our CTNs and nurse may have failed to fully address medications or review the importance of using a pillbox for managing medications.

The HP-CMA CE was based on the program developed by Possin and colleagues that took place at University of California, San Francisco and University of Nebraska Medical Center. However, due to the nature of a non-academic, clinical environment, our CE implementation differed in terms of its fidelity to the previous described program in a variety of ways. Firstly, we had no formal inclusion criteria beyond the fact that patients had to be aged > 45 and have a history of MCI or dementia. We did not require PWDs to be enrolled in Medicare or Medicaid, and dyads did not need to complete formal consent to receive the care. The HP-CMA “dose” of phone contacts with the CTN was less than that of the original CE model, which was primarily due to the higher caseloads at our site and relatively milder stage of dementia. Furthermore, whereas prior versions of the CE included an embedded pharmacist, the HP-CMA did not have the ability to provide the CE’s formal medication review service on every patient [12] and instead deferred medication management questions to either the nurse or participating neurologist. In addition, data relating to outcomes and survey data were obtained by mailing information either traditionally or via email to participating dyads whereas UCSF research coordinators conducted formalized telephone surveys with participants. We suspect this factor impacted response rate to follow-up assessments and potentially data accuracy. Finally, we did not include the QoL-AD outcome that assessed PWD quality of life as rated by the caregiver. In the published CE randomized clinical trial, caregiver QoL-AD of the PWD declined in both treatment and usual care groups but the decline was more pronounced in usual care arm. Since caregiver rated QoL declines with progressive dementia, we did not feel that assessment of this variable would provide meaningful information in the absence of a control population of dyads.

Strengths of this investigation include the largest cohort outside of an academic institution to be enrolled into the CE. Furthermore, this program was able to target 19% of dyads living within rural Minnesota and Wisconsin-based communities with limited access to care. Limitations beyond what was described above include the lack of comparison to a control group. The extensive staff turnover may have further impacted continuity of care. It should also be noted that longitudinal data from follow-up surveys was available for only 30% of participants, and the CE initiative was performed in a specialized dementia clinic, both factors that may impact the generalizability of this study’s results. Application of the CE in primary care would be expected to have the broadest impact on dyad care and outcomes, but would have required extensive adaptive work that was beyond the infrastructure of our current CE team. Data relating to hospitalization and ED visitation was extracted from the Epic EMR, which only captured clinical information from within the HealthPartners system as well as from outside major hospital systems through “care everywhere.”

Our experience applying the CE within the HealthPartners integrated medical system indicates that this program can be successfully integrated into purely clinical settings. Next steps are to participate in a clinical investigation to evaluate healthcare utilization outcomes across multiple healthcare organizations by comparing EHR data between CE and non-CE enrolled dyads. In addition, we hope to evaluate the application of the CE in primary care clinics where the majority of PWDs are managed and where there is the greatest demand for care management services.

## Data Availability

All data generated or analyzed during this study are included in this published article.
